# Effect of Anodal Transcranial Direct Current Stimulation Combined With Cognitive Training for Improving Cognition and Language Among Children With Cerebral Palsy With Cognitive Impairment: A Pilot, Randomized, Controlled, Double-Blind, and Clinical Trial

**DOI:** 10.3389/fped.2021.713792

**Published:** 2021-08-25

**Authors:** Eun Jae Ko, Mi Jin Hong, Eun Jung Choi, Jin Sook Yuk, Mi Sun Yum, In Young Sung

**Affiliations:** ^1^Department of Rehabilitation Medicine, Asan Medical Center, University of Ulsan College of Medicine, Seoul, South Korea; ^2^Department of Rehabilitation Medicine, Konyang Medical Center, University of Konyang College of Medicine, Daejeon, South Korea; ^3^Department of Rehabilitation Medicine, Seongnam Citizens Medical Center, Seongnam, South Korea; ^4^Department of Rehabilitation Medicine, Asan Medical Center, Seoul, South Korea; ^5^Department of Pediatrics, Asan Medical Center Children's Hospital, University of Ulsan College of Medicine, Seoul, South Korea

**Keywords:** cerebral palsy, cognitive dysfunction, language, transcranial direct current stimulation, child

## Abstract

About 30–45% of cerebral palsy (CP) patients have cognitive impairment. Previous studies showed the evidence that transcranial direct current stimulation (tDCS) may have some benefits in attention-deficit/hyperactivity disorder, autism spectrum disorder, and motor development in CP. The aim of this study is to evaluate the effect of tDCS on cognition, language, and activities of daily living (ADL) among children with CP with cognitive impairment. It was a pilot, randomized, controlled, double-blind, clinical trial in a tertiary pediatric hospital, and 13 children with CP and a cognitive age under 42 months were enrolled. tDCS group (*n* = 8) had active tDCS and cognitive training (20 min/session, total 20 sessions, for 12 weeks) and sham group (*n* = 5) had sham tDCS and cognitive training. Primary outcome was the Bayley Scales of Infant Development II (BSID II). Secondary outcomes were the Pediatric Evaluation of Disability Inventory (PEDI), the Laboratory Temperament Assessment Battery (Lab-TAB), the Early Childhood Behavior Questionnaire (ECBQ), the Korean version of MacArthur–Bates Communicative Development Inventories (M-B CDI-K), the Sequenced Language Scale for Infants (SELSI) and the Preschool Receptive-Expressive Language Scale (PRES). After intervention, the tDCS group showed significant improvements in all measurements (*p* < 0.05) except the M-B CDI-K (grammar), whereas the sham group only showed significant improvements in the Lab-TAB (manipulation domain), the ECBQ (attentional shifting), and the M-B CDI-K (comprehension). The between-group differences in the degree of post-intervention improvement were not statistically significant. The degree of improvement was associated with better baseline cognitive function and younger age (*p* < 0.05). There were no major adverse events after tDCS. The combined application of tDCS and cognitive training was feasible and associated with improvements in cognitive function, ADL, and language among children with CP with cognitive impairment. However, considering that it is a pilot study, further larger-scale systematic investigation is needed.

**Clinical Trial Registration:** The trial was registered in the Clinical Research Information Service database, identifier: KCT0003023.

## Introduction

Cerebral palsy (CP) is characterized by early insults to the developing brain, resulting in ongoing problems in movements or postures that limit the performance of activities of daily living (ADL) (1). CP is usually accompanied by motor impairments; however, sensory and perception disturbances, global or specific cognitive impairment, communication disorders, behavioral problems, and seizures are also commonly present (2). About 30–45% of CP patients have cognitive impairment (3, 4), which can manifest as altered information processing, attention impairment, decreased executive function, and memory and language (5). Many cognitive treatments have been tried, including traditional occupational therapy targeting cognition, computer-based working memory training (6), and virtual reality (5).

Early interventions are known to be beneficial for alleviating motor impairments in children with CP, however there are less rationale of the effect of cognitive training (7). There are some evidences of cognitive orientation to occupational performance (CO-OP) (8), literacy interventions using communication devices (9), and GAME intervention (Goal—Activity—Motor enrichment) (10) in improving cognition in children with CP, however, it is still challenging. In clinical field, cognitive training focusing on attention, memory, executive function, and perception–motor function is usually performed in children with CP.

Non-invasive brain stimulation is widely used due to its potential to modulate cortical excitability and plasticity, and transcranial magnetic stimulation (TMS) and transcranial direct current stimulation (tDCS) are the most frequently used forms of non-invasive brain stimulation (11). In TMS, a fluctuating extracranial magnetic field induces intracranial electrical currents in the cortex, whereas in tDCS, scalp electrodes induce constant electrical currents to the brain (12). Compared with TMS, tDCS is advantageous because it can deliver constant current for a longer time, it delivers a lower current that can change the cortical excitability, there is no sound or flinch of muscles, it can be easily used with other rehabilitation treatments, and it is conducive to performing sham-controlled double-blind studies (13). In a systematic review (14) of adverse effects associated with tDCS among children and adolescents, tingling (11.5%), itching (5.8%), redness (4.7%), and scalp discomfort (3.1%) were reported, all of which were mild and transient.

Previous studies have investigated the effects of tDCS among children with refractory epilepsy, attention-deficit/hyperactivity disorder (ADHD), and autism spectrum disorder (ASD) (15, 16). Several randomized placebo-controlled trials (17–19) have suggested beneficial effects of tDCS for treating CP; however, the tDCS used in these studies targeted the primary motor cortex and resulted in improvements in balance, spasticity, and functional performance. To the best of our knowledge, no published studies have investigated the effects of tDCS on cognition or language among children with CP, who have early structural defect in the developing brain, inducing abnormal network.

Therefore, this study aimed to evaluate the effects of tDCS on cognition, language, and ADL among children with CP with cognitive impairment.

## Materials and Methods

### Study Design and Participants

**Figure 1:**
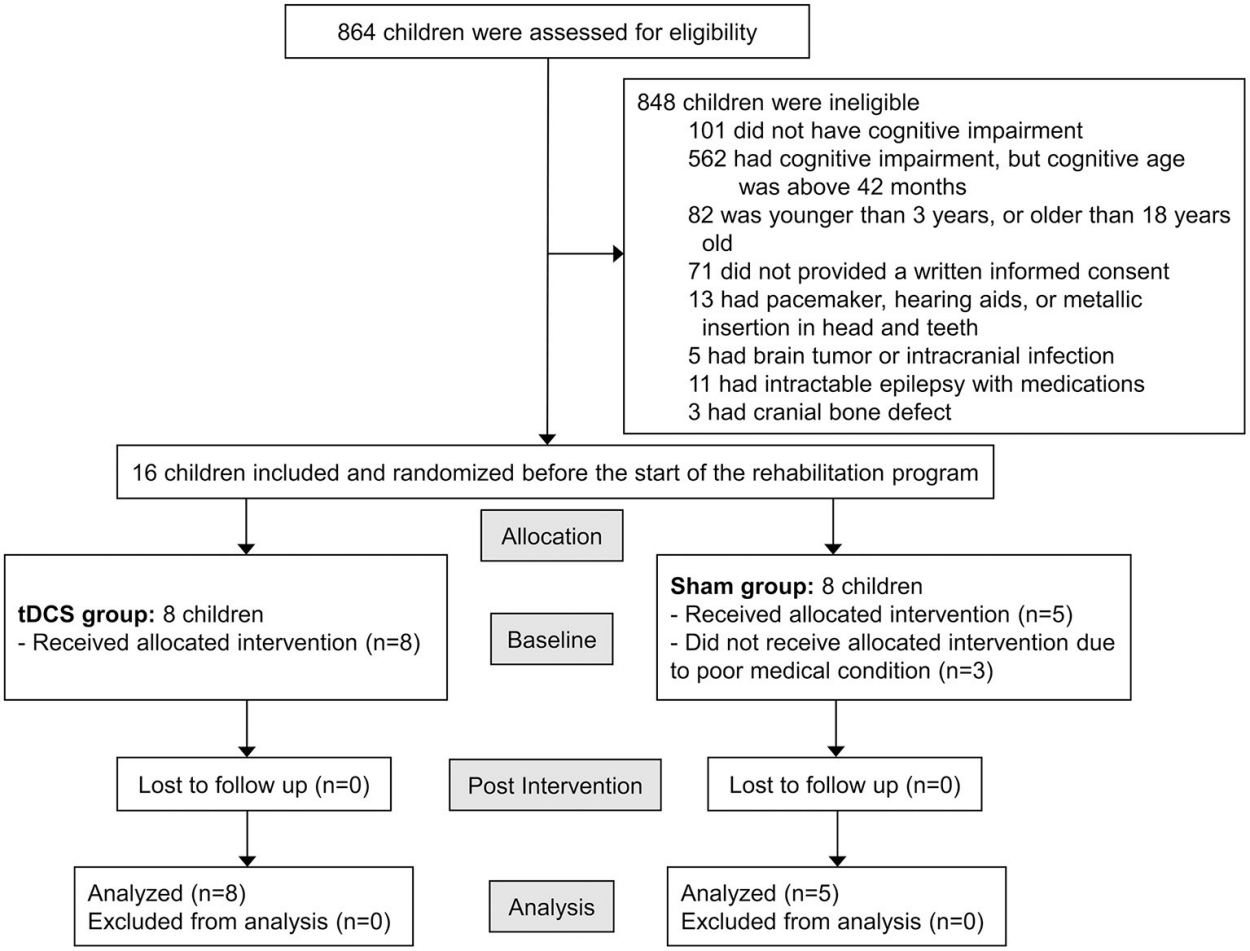
Participant enrollment flow diagram.

This pilot study was approved by the Ethical Committee of Asan Medical Center (ref number: 2018-0150). The parents or legal guardians of all participants provided written informed consent before data collection began. The trial was registered in the Clinical Research Information Service database (ref number: KCT0003023). Figure 1 shows the study enrollment flowchart.

Children who visited the outpatient clinic of the Pediatric Rehabilitation Medicine Division at Asan Medical Center from July 2018 through January 2019 were assessed for inclusion in the study according to the following inclusion criteria: (1) children with CP, diagnosed by pediatric physiatrists; (2) children with cognitive impairment and a cognitive age under 42 months, as assessed using the Bayley Scales of Infant Development II (BSID II); (3) children whose actual age was between 3 and 18 years; (4) children who had not changed medications for cognition, language, and physical function, and who had not changed the number of rehabilitation programs in which they participated; and (5) children whose caregivers provided written informed consent. Exclusion criteria were as follows: (1) children with pacemakers, hearing aids, or metallic insertions in head or teeth; (2) children with brain tumors or intracranial infections; (3) children who had epilepsy intractable with medications; (4) children who had cranial bone defects; and (5) children with neurodegenerative diseases.

### Randomization and Masking

Children were randomized using a computer-generated random number list and allocated to either a tCDS group (active tDCS and cognitive training) or sham group (sham tDCS and cognitive training). Randomization was carried out by someone other than the person who carried out recruitment, tDCS, and cognitive training. The randomization process used a random table with a ratio of 1:1. All children, caregivers, the two fixed occupational therapists involved in the evaluation, and the two investigators who conducted the study, were blinded to the treatment allocation.

### Intervention

The tDCS group underwent cognitive training with active tDCS, and the sham group underwent cognitive training with sham tDCS for 20 min per day and a total of 20 sessions. The DC-Stimulator Plus (neuroConn, Germany) was used for tDCS. This device was approved by the Ministry of Food and Drug Safety in Korea. The anode electrode (5 × 5 cm) was placed over the more involved side (right or left) of the dorsolateral prefrontal cortex (DLPFC), and the cathode electrode was placed over the contralateral supraorbital region, with the use of the Omni-Lateral-Electrode system (20). In other words, the left DLPFC was selected for children with CP in whom the left cerebral hemisphere was more involved than the right hemisphere, and the right DLPFC was selected for children with CP in whom the right hemisphere was predominantly affected. In the tDCS group, 1 mA of direct-current stimulation was administered. The participants of this study underwent a similar protocol of tDCS with the previous studies (21) discussing the effect of tDCS targeting cognition. Furthermore, the first 10 sessions took place daily, except for weekends, and the remaining 10 sessions took place once a week, until week 12, which is the similar to the previous study (22). The tDCS protocol used in this trial had more sessions than in earlier trials because recent studies have suggested that more sessions could produce greater clinical effects (23).

The same protocol was used for the sham group; however, the current was turned off automatically after 40 s in patients receiving sham tDCS by the devices that were programmed to deliver sham stimulation according to the randomization code.

Trained occupational therapists provided cognitive training for participants in both groups. Cognitive training was performed for 20 min per day and a total of 20 sessions: the first 10 sessions took place daily, except for weekends, and the remaining 10 sessions took place once a week. Cognitive training focused on different cognitive domains including attention, memory, executive function, perception–motor function, and eye-hand coordination. Cognitive training was performed using blocks, toys, puzzles, color matching, memory games, finding hidden objects, and tracing.

### Outcome Measurements

Children were evaluated before and after receiving therapeutic interventions for 12 weeks. The primary outcome was assessed using the BSID II (24), and secondary outcomes were evaluated using the Pediatric Evaluation of Disability Inventory (PEDI) (25), the Laboratory Temperament Assessment Battery (Lab-TAB) (26), the Early Childhood Behavior Questionnaire (ECBQ) (27), the Korean version of the MacArthur–Bates Communicative Development Inventories (M-B CDI-K) (28), and the Sequenced Language Scale for Infants (SELSI) (29) or the Preschool Receptive-Expressive Language Scale (PRES) (30). The BSID II, PEDI, and Lab-TAB were evaluated by experienced occupational therapists, and SELSI and PRES were evaluated by experienced speech therapists. ECBQ and M-B CDI-K were evaluated by parents. The occupational and speech therapists, and parents were all unaware of the trial group assignments.

The BSID II (24) is the most widely used tool for assessing developmental progress in Mental and Motor scales between 1 and 42 months of chronological age, but it is also applicable for children over 42 months of chronological age with developmental delay. In this study, only the Mental scale was used. Cognitive age was determined using the BSID II raw mental score and the table in the BSID II manual titled, “Raw score equivalents for developmental ages for the mental and motor scales.” When the difference between the cognitive age and the chronological age was more than 6 months, this was considered cognitive impairment. The PEDI (25) is an instrument that measures independence in daily living and covers daily activities in self-care, mobility, and social functioning among children between 6 months and 7.5 years of age. The Lab-TAB (26) assesses infant responses to stimuli that elicit emotional or behavioral reactivity, and only one dimension of “interest/persistence” was used to assess attention, which is assessed with either a block play paradigm or a bead play paradigm, depending on the ability of the child. The ECBQ (27) is a parent-report measure that evaluates behavior during early childhood between the ages of 18 and 36 months, however, it is also applicable to children over 36 months of chronological age with developmental delay. The full version measures 18 discrete traits, but only the “attentional focusing” and “attentional shifting” traits were used in this study to measure attention. The M-B CDI-K (28) is a simple screening test for language developmental delay, and it consists of parent reports on early lexicon and other communicative/language behaviors. The SELSI (29) or the PRES (30) was used to evaluate language abilities, and receptive and expressive language ages were determined as follows: the former was for children under the age of 3 years and the latter for preschoolers above the age of 3 years. The SELSI was conducted in some children whose language levels were inadequate for assessment using the PRES.

Additionally, data on chronological age, sex, Gross Motor Function Classification System (GMFCS) level (31), and the side of anodal stimulation were collected.

### Adverse Event Questionnaire

To assess adverse events, all caregivers completed a questionnaire that queried symptoms and side effects after tDCS, which included headache, neck pain, scalp pain, numbness, tingling, itching, burning sensation, erythema, sleepiness, difficulties concentrating, and mood changes.

### Statistical Analysis

Data were analyzed using SPSS for Windows, version 21.0 (IBM Corp., Armonk, NY, USA). Means, standard deviations, median, and interquartile range were calculated with a threshold for statistical significance set at *p* < 0.05. To compare the baseline characteristics of the two groups, the Mann–Whitney *U*-test, Fisher's exact test, and the chi-square test were used, as appropriate. The Wilcoxon signed-rank test was used to compare the pre- and post-treatment measurements of the two groups, and the Mann–Whitney *U*-test was used to compare changes in measurements between the two groups. Logistic regression analysis was used to determine independent factors associated with improvements in outcomes in the tDCS group. Additional analysis using the Wilcoxon signed-rank test and the Mann–Whitney *U*-test was performed after matching the number of subjects and GMFCS levels between the two groups.

## Results

### Baseline Characteristics of the Children With CP

Eight children were in the tDCS group and five were in the sham group. Baseline characteristics of the tDCS and sham groups are given in Table 1. The mean chronological age of the tDCS group was 70.1 months (median 63, range 37–108), with three males and five females, and the mean age of the sham group was 84.0 (median 74, range 48–120), with three males and two females. There were no significant differences between the two groups in terms of baseline characteristics, including age, sex, GMFCS level, side of anodal stimulation, and all outcome measures.

**Table 1:** Comparison of baseline characteristics between the tDCS and sham groups.

	**tDCS group** (***n*****= 8)**	**Sham group** (***n*****= 5)**
Age (months)	70.1 (27.3)	84.0 (34.1)
Sex (Male:Female)	3:5	3:2
GMFCS level (I:II:III:IV:V)	0:3:1:3:1	1:0:1:2:1
Side of anodal stimulation (Right:Left)	6:2	3:2
Mental scale of BSID II	120.5 (36.7)	138.0 (16.9)
PEDI (Self-care)	27.3 (17.5)	30.6 (16.3)
PEDI (Mobility)	19.4 (14.5)	20.0 (17.7)
PEDI (Social function)	25.1 (18.7)	29.2 (11.0)
Lab-TAB (Observation)	31.4 (18.4)	31.2 (13.8)
Lab-TAB (Manipulation)	27.9 (17.9)	19.4 (14.2)
ECBQ (Attentional focusing)	36.4 (14.4)	37.4 (11.0)
ECBQ (Attentional shifting)	47.8 (18.1)	42.4 (10.6)
M-B CDI-K (Word)	162.3 (284.5)	245.8 (222.2)
M-B CDI-K (Comprehension)	313.6 (232.4)	383.8 (177.1)
M-B CDI-K (Grammar)	18.3 (33.8)	19.0 (27.9)
Speech comprehension	28.5 (19.1)	31.6 (11.8)
Speech expression	25.8 (21.4)	24.4 (11.4)

*Values are presented as mean (SD). BSID II, Bayley Scales of Infant Development II; PEDI, Pediatric Evaluation of Disability Inventory; Lab-TAB, Laboratory Temperament Assessment Battery; ECBQ, Early Childhood Behavior Questionnaire; M-B CDI-K, Korean version of MacArthur–Bates Communicative Development Inventories*.

*p > 0.05 by Mann–Whitney U-test, Fisher's exact test, or chi-Square test*.

### Comparisons of Outcomes Within and Between the Two Groups

**Figure 2:**
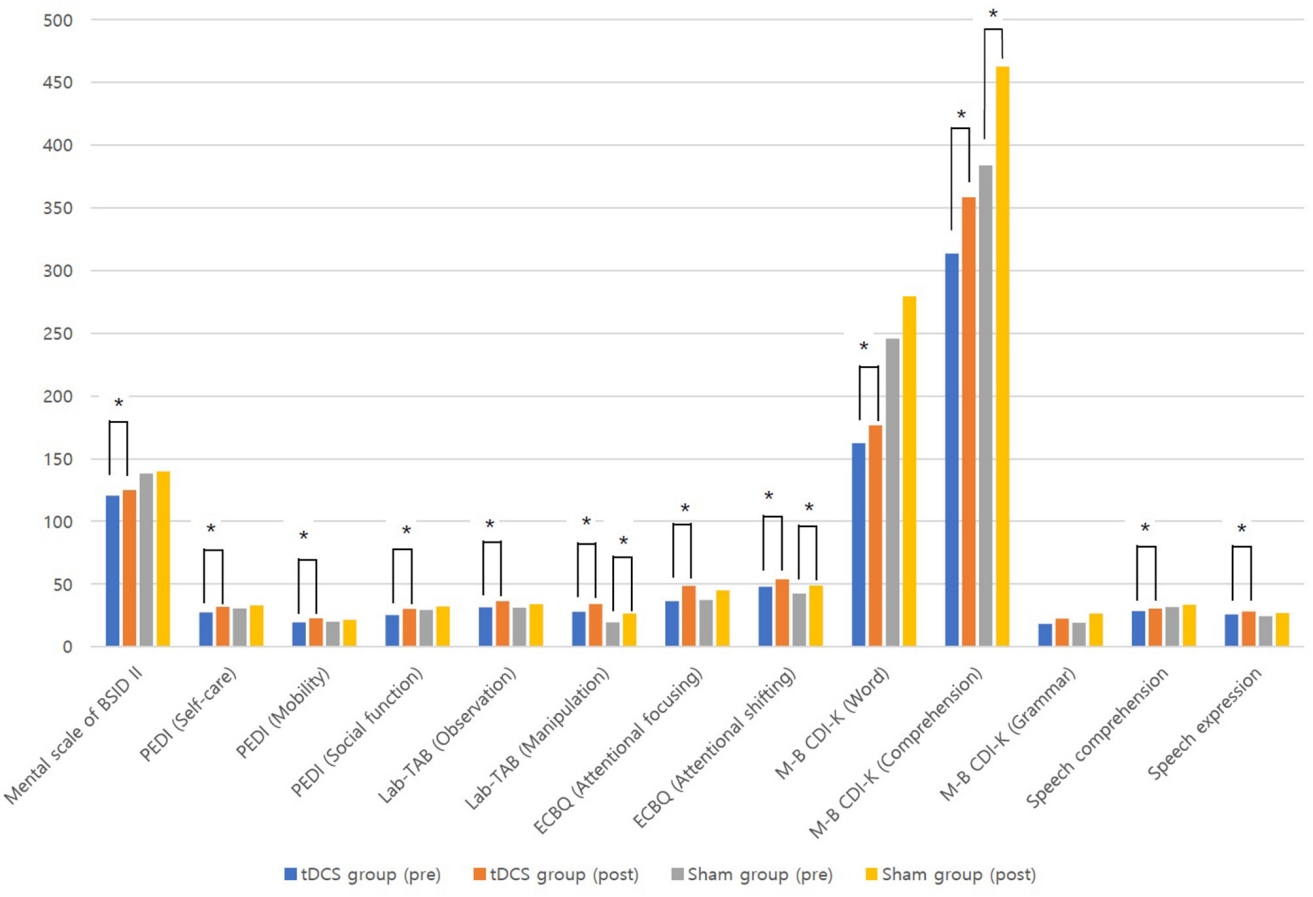
Comparisons of outcomes before and after treatment in tDCS and sham groups. *p < 0.05 by Wilcoxon signed-rank tes.

Table 2 shows comparisons of outcomes within the two groups. After 12 weeks of intervention, the tDCS group showed significant improvements in all measurements (*p* < 0.05) except the grammar domain of the M-B CDI-K, whereas the sham group only showed significant improvements in the manipulation domain of the Lab-TAB, the attentional shifting domain of the ECBQ, and the comprehension domain of the M-B CDI-K (Figure 2). When comparing the degree of post-intervention improvement between the groups, the differences were not statistically significant (*p* > 0.05; Table 3).

**Table 2:** Comparisons of outcomes before and after treatment in each group.

	**tDCS group (***n*** = 8)**			**Sham group (***n*** = 5)**		
	**Before treatment**	**After treatment**	***p***	**Before treatment**	**After treatment**	***p***
Mental scale of BSID II	120.5 (36.7)	125.0 (37.7)	0.02^*^	138.0 (16.9)	140.0 (17.0)	0.06
PEDI (Self-care)	27.3 (17.5)	31.8 (18.9)	0.03^*^	30.6 (16.3)	33.0 (15.3)	0.07
PEDI (Mobility)	19.4 (14.5)	22.6 (13.9)	0.03^*^	20.0 (17.7)	21.6 (17.4)	0.11
PEDI (Social function)	25.1 (18.7)	30.1 (18.9)	0.02^*^	29.2 (11.0)	32.2 (11.7)	0.07
Lab-TAB (Observation)	31.4 (18.4)	36.4 (18.6)	0.03^*^	31.2 (13.8)	33.8 (13.4)	0.08
Lab-TAB (Manipulation)	27.9 (17.9)	34.0 (17.3)	0.03^*^	19.4 (14.2)	26.6 (13.1)	0.04^*^
ECBQ (Attentional focusing)	36.4 (14.4)	48.5 (18.1)	0.04^*^	37.4 (11.0)	45.0 (18.8)	0.22
ECBQ (Attentional shifting)	47.8 (18.1)	53.9 (20.6)	0.04^*^	42.4 (10.6)	48.6 (11.8)	0.04^*^
M-B CDI-K (Word)	162.3 (284.5)	176.5 (285.8)	0.03^*^	245.8 (222.2)	279.4 (245.6)	0.11
M-B CDI-K (Comprehension)	313.6 (232.4)	358.5 (237.1)	0.01^*^	383.8 (177.1)	462.6 (169.5)	0.04^*^
M-B CDI-K (Grammar)	18.3 (33.8)	22.5 (32.9)	0.07	19.0 (27.9)	26.6 (13.8)	0.11
Speech comprehension	28.5 (19.1)	30.4 (19.8)	0.02^*^	31.6 (11.8)	33.4 (11.2)	0.11
Speech expression	25.8 (21.4)	28.1 (20.8)	0.04^*^	24.4 (11.4)	27.0 (12.1)	0.07

*Values are presented as mean (SD). BSID II, Bayley Scales of Infant Development II; PEDI, Pediatric Evaluation of Disability Inventory; Lab-TAB, Laboratory Temperament Assessment Battery; ECBQ, Early Childhood Behavior Questionnaire; M-B CDI-K, Korean version of MacArthur–Bates Communicative Development Inventories*.

* *p < 0.05 by Wilcoxon signed-rank test*.

**Table 3:** Comparisons of improvements in outcomes between the two groups.

	**tDCS group** (***n*****= 8)**	**Sham group** (***n*****= 5)**	***p***
Mental scale of BSID II	4.5 (4.6)	2.0 (1.4)	0.22
PEDI (Self-care)	4.5 (3.9)	2.4 (1.8)	0.52
PEDI (Mobility)	3.3 (2.5)	1.6 (1.8)	0.22
PEDI (Social function)	5.0 (4.0)	3.0 (2.0)	0.44
Lab-TAB (Observation)	5.0 (4.1)	2.6 (2.5)	0.35
Lab-TAB (Manipulation)	6.1 (6.4)	7.2 (3.0)	0.52
ECBQ (Attentional focusing)	12.1 (14.9)	7.6 (12.9)	0.52
ECBQ (Attentional shifting)	6.1 (6.7)	6.2 (4.6)	0.83
M-B CDI-K (Word)	14.3 (20.6)	33.6 (38.6)	0.62
M-B CDI-K (Comprehension)	44.9 (52.0)	78.8 (66.4)	0.22
M-B CDI-K (Grammar)	4.3 (9.7)	7.6 (8.7)	0.52
Speech comprehension	1.9 (1.7)	1.8 (2.5)	0.72
Speech expression	2.4 (3.0)	2.6 (2.4)	0.72

*Values are presented as mean (SD). BSID II, Bayley Scales of Infant Development II; PEDI, Pediatric Evaluation of Disability Inventory; Lab-TAB, Laboratory Temperament Assessment Battery; ECBQ, Early Childhood Behavior Questionnaire; M-B CDI-K, Korean version of MacArthur–Bates Communicative Development Inventories*.

*p > 0.05 by Mann–Whitney U-test*.

### Factors Associated With Outcome Improvements in the tDCS Group

Table 4 shows the factors associated with improved outcomes in the tDCS group (*n* = 8). The degree of improvement in the self-care domain of the PEDI was associated with higher baseline scores in the Mental scale of the BSID II, the social function domain of the PEDI, the observation and manipulation domains of the Lab-TAB, and the attentional shifting domain of the ECBQ. The degree of improvement in the attentional focusing domain of the ECBQ was associated with a higher baseline score in the observation domain of the Lab-TAB. The degree of improvement in the word domain of the M-B CDI-K was associated with a higher baseline score in the manipulation domain of the Lab-TAB. Furthermore, the degree of improvement in speech expression was associated with younger age.

**Table 4:** Independent factors associated with improved outcomes in the tDCS group (*n* = 8).

**Outcome measure**	**Independent factors**	**B**	**SE**	***p***
PEDI (Self-care)	Baseline Mental scale of BSID II	0.09	0.03	0.02^*^
	Baseline PEDI (Social function)	0.15	0.06	0.04^*^
	Baseline Lab-TAB (Observation)	0.18	0.05	0.01^*^
	Baseline Lab-TAB (Manipulation)	0.17	0.06	0.03^*^
	Baseline ECBQ (Attentional shifting)	0.17	0.06	0.03^*^
ECBQ (Attentional focusing)	Baseline Lab-TAB (Observation)	0.58	0.23	0.045^*^
M-B CDI-K (Word)	Baseline Lab-TAB (Manipulation)	0.88	0.30	0.03^*^
Speech expression	Age	−0.08	0.03	0.04^*^

*Data analyzed with univariate logistic regression analysis, enter method*.

*SE, standard error; BSID II, Bayley Scales of Infant Development II; PEDI, Pediatric Evaluation of Disability Inventory; Lab-TAB, Laboratory Temperament Assessment Battery; ECBQ, Early Childhood Behavior Questionnaire; M-B CDI-K, Korean version of MacArthur–Bates Communicative Development Inventories*.

* *p < 0.05 by logistic regression analysis*.

### Additional Analysis After Matching Samples

When the number of subjects and GMFCS levels between the two groups were matched, 4 children (1: GMFCS level III, 2: GMFCS level IV, 1: GMFCS level V) were included in the tDCS and sham groups respectively. The median chronological age of the tDCS group was 87.0 months, with 2 males and 2 females. and the median age of the sham group was 89.0, with 3 males and 1 female. There were no significant differences between the two groups in baseline characteristics. Table 5 shows comparisons of outcomes within and between the two groups after matching samples. There was no significant change of outcome measurements within and between groups.

**Table 5:** Subgroup analysis between the groups after matching.

	**tDCS group** (***n*** **= 4)**			**Sham group** (***n*** **= 4)**		
	**Before** **treatment**	**After** **treatment**	**Change value**	**Before** **treatment**	**After** **treatment**	**Change value**
Mental scale of BSID II	130.0 (77.5)	131.5 (80.8)	2.0 (2.8)	140.5 (12.5)	142.5 (14.0)	2.0 (1.0)
PEDI (Self-care)	22.5 (20.8)	27.5 (27.5)	5.0 (6.8)	33.5 (18.5)	27.0 (15.3)	2.5 (1.5)
PEDI (Mobility)	11.5 (7.0)	15.0 (11.8)	2.5 (3.8)	13.0 (7.0)	14.5 (10.0)	2.0 (2.5)
PEDI (Social function)	31.5 (39.5)	36.0 (42.0)	2.0 (3.0)	28.5 (15.5)	31.5 (17.3)	4.0 (0.8)
Lab-TAB (Observation)	35.5 (19.0)	44.0 (21.0)	6.5 (4.5)	36.0 (15.0)	38.5 (9.8)	3.0 (1.8)
Lab-TAB (Manipulation)	22.0 (20.8)	35.5 (20.8)	8.0 (11.0)	16.5 (21.0)	23.5 (18.0)	7.0 (3.0)
ECBQ (Attentional focusing)	33.0 (22.3)	41.5 (23.8)	8.5 (6.0)	44.5 (7.0)	43.5 (13.3)	2.0 (12.3)
ECBQ (Attentional shifting)	44.5 (38.3)	53.0 (30.3)	4.0 (7.5)	44.0 (15.3)	47.0 (12.3)	8.0 (7.0)
M-B CDI-K (Word)	310.0 (611.8)	324.0 (628.8)	4.0 (7.0)	165.5 (299.5)	216.5 (364.0)	42.5 (14.3)
M-B CDI-K (Comprehension)	368.5 (515.3)	391.0 (510.5)	23.5 (11.3)	346.5 (217.0)	450.0 (215.8)	87.5 (71.8)
M-B CDI-K (Grammar)	36.0 (72.5)	36.5 (73.3)	0.0 (0.3)	13.0 (35.8)	23.5 (53.3)	3.5 (10.5)
Speech comprehension	35.5 (36.5)	36.0 (38.0)	1.0 (1.0)	33.5 (18.5)	33.5 (18.3)	0.5 (1.3)
Speech expression	35.5 (43.5)	36.5 (45.0)	0.0 (0.5)	24.5 (20.5)	26.0 (20.8)	1.5 (1.8)

*Values are presented as median (IQR). BSID II, Bayley Scales of Infant Development II; PEDI, Pediatric Evaluation of Disability Inventory; Lab-TAB, Laboratory Temperament Assessment Battery; ECBQ, Early Childhood Behavior Questionnaire; M-B CDI-K, Korean version of MacArthur–Bates Communicative Development Inventories*.

*p > 0.05 by Wilcoxon signed-rank test*.

### Adverse Events and Safety of tDCS

Thirteen caregivers of the children in both groups completed the questionnaire regarding symptoms and side effects after active or sham tDCS. Only one caregiver replied that the child's mood seemed to change to a moderate degree after tDCS. The rest of the 12 caregivers replied that there were no symptoms or side effects observed after tDCS, including headache, neck pain, scalp pain, numbness, tingling, itching, burning sensation, erythema, sleepiness, or difficulties concentrating.

## Discussion

The results of this study indicate that anodal tDCS and cognitive training for 20 min per session for 12 weeks, for a total of 20 sessions, significantly improved cognitive function (as proven by BSID II, Lab-TAB, ECBQ), ADL (as proven by PEDI), and language (as proven by M-B-CDI-K, SELSI, and PRES) among children with CP with cognitive impairment, without serious adverse events. Only the grammar domain of M-B-CDI-K was not significantly improved in the tDCS group, and this can be explained by the order of difficulty in language; grammar domain usually improves later than the word and the comprehension domain. However, sham group (sham tDCS and cognitive training) also improved the manipulation domain of the Lab-TAB, the attentional shifting domain of the ECBQ, and the comprehension domain of the M-B CDI-K. We consider these improvements the results of 12 weeks of cognitive training in the sham group. When comparing the degree of post-intervention improvement between the two groups, the tDCS group showed more improvements than the sham group. However, these differences were not statistically significant, which may be due to the small number of patients included, and impaired brain structure of the children with CP. Although the mechanism of combining tDCS and cognitive training together is not proved yet, tDCS stimulating the DLPFC, which is the main cortex related with working memory (32), may excite the cognitive process and functional connectivity in the brain, as shown in a previous study (33) of TMS in children with CP. Accordingly, tDCS may maximize the effect of cognitive training of children with CP. In terms of improved outcomes in the tDCS group, the effects of tDCS were more prominent among patients with better baseline cognitive function and younger patients, as shown in Table 4.

When the number of subjects and GMFCS levels between the two groups were matched, there was no significant change of outcome measurements within and between groups. Since there were too small number of children included in each group (*n* = 4), further study is needed to prove the effect of the tDCS with matched controls.

The first animal experiments regarding tDCS were conducted in the early 1960s; these experiments showed polarity dependent direct current–mediated modulation of cortical neuronal activity (34), but the interest in brain stimulation decreased afterward. The application of tDCS in humans started at the beginning of the 21st century (35). tDCS provokes sub-threshold modulation of neuronal excitability without depolarizing action potentials. This modulation is polarity-dependent toward depolarization after anodal stimulation (excitatory) and toward hyperpolarization after cathodal stimulation (inhibitory), leading to transient changes in the resting membrane potential (16). The cumulative effect of longer stimulation results in polarity-dependent facilitation or inhibition of spontaneous neuronal firing (36), which is considered neuromodulatory. However, the tDCS parameters are still controversial, and there is still a lack of knowledge about the late impact of tDCS on the developing brain.

Previous studies have investigated the effects of anodal tDCS on cognition, and these have usually focused on working memory (21). Working memory is associated with a variety of higher-order cognitive abilities, including selective attention, reading comprehension, reasoning, and decision-making (37–39). The DLPFC (Brodmann area 9/46), with its large neuroanatomical connections to numerous cortical and subcortical structures, is strongly associated with working memory (32), and consequently, the DLPFC has been selected as the target region for the effect of tDCS on cognition or working memory. This region can be stimulated by positioning the anode over either the F3 (left DLPFC) or F4 (right DLPFC) regions on the scalp according to the international 10–20 system for electrode placement (40). Berryhill and Jones (41) investigated the effects of anodal tDCS on cognition by stimulating left or right DLPFC and found that tDCS was uniformly beneficial on both sides. Since anodal tDCS results in depolarization of the membrane potential of neural cells and facilitates spontaneous neuronal firing, the more involved side of the DLPFC was selected for anodal stimulation to activate the region of the brain in this study, which significantly improved cognitive function among children with CP and cognitive impairment. Improved cognition may facilitate improved language function and proficiency in ADL.

In this study, anodal tDCS was concurrently applied with cognitive training in the tDCS group, and this seemed to be more effective for improving cognition than cognitive training alone. In fact, tDCS-induced plasticity is highly dependent on the state of the subject during stimulation. Liao et al. (42) showed that tDCS both before and during mirror therapy resulted in better motor function than tDCS alone among chronic stroke patients. However, studies comparing the effects of tDCS alone and concurrent or sequential use of tDCS and cognitive training are scarce so far, and future studies are warranted.

There are not many published studies discussing the effects of tDCS on cognitive function among children; however, some studies have investigated the intervention in children with ADHD. Bandeira et al. (43) conducted a pilot study to investigate the effects of anodal tDCS (anode over left DLPFC, cathode over right supraorbital, 2 mA, 30 min, five sessions on consecutive days) among nine children with ADHD (mean age 11.1 years, range 7–15) when performing a card matching game for training. They observed improvements in selective attention and a reduction of errors in an inhibitory control task; however, there was no control group. In another study by Soltaninejad et al. (44), anodal tDCS (anode over left DLPFC, cathode over right supraorbital, 1.5 mA, 15 min, single session) was applied for 20 children with ADHD (mean age 16.4 years, range 15–17) and was associated with improvements in correct go-answers in a go/no-go task compared with sham tDCS. Additionally, cathodal tDCS (cathode over left DLPFC, anode over right supraorbital, 1.5 mA, 15 min, single session) improved inhibition accuracy in the go/no-go task compared with sham tDCS, indicating improvement in inhibitory control. One of the most probable theories of the neural basis of ADHD has focused on deficient inhibitory control, which leads to executive dysfunction (45, 46). The neuroanatomic substrate of inhibitory control is associated with the basal ganglia–thalamocortical circuits (47), and this was confirmed by structural and functional ADHD neuroimaging studies (48–50). A potential benefit of tDCS in ADHD could be improved behavioral inhibition by affecting this circuit, resulting in improved attention and memory.

Some studies have also discussed the effects of tDCS on cognitive function among children with ASD. Amatachaya et al. (51) conducted a randomized, double-blind crossover trial involving 20 children with ASD (mean age 6.4 years, range 5–8). The children received both active and sham tDCS (anode over left DLPFC, cathode over right shoulder, 1 mA, 20 min, five sessions on consecutive days) in different orders with a 4-week washout period. Active tDCS resulted in more improvements in the sensory and cognitive awareness subscales of the Autism Treatment Evaluation Checklist than the sham group. In a study by Schneider and Hopp (52), tDCS (anode over left DLPFC, cathode right supraorbital, 2 mA, 30 min, single session) involving 10 minimally verbal children with ASD (mean age 9.8, range 6–21) resulted in increased syntax comprehension. The pathophysiology of ASD is not fully understood (53), but ASD patients showed a decreased volume (54) and hypoactivation (55, 56) of the left cerebral hemisphere relative to the right hemisphere. Atypical rightward asymmetry may be a pervasive feature of functional brain organization in ASD, affecting sensorimotor, as well as higher cognitive, domains (56). Furthermore, disturbed cell migration, abnormal synaptic maturation, and decreased brain connectivity may contribute to ASD (57), and lower electroencephalography (EEG) alpha activity was shown in children with ASD relative to healthy children (58). In one study, anodal tDCS over the left DLPFC increased alpha frequency with an improvement of ASD symptoms, and this may explain the therapeutic mechanism of tDCS for ASD (59).

There are few published articles on the use of tDCS in the pediatric population, and this study is perhaps the first to test the potential benefits of this technique for children with CP through stimulation of the DLPFC. The results confirm the safety and tolerability of this technique and the potential benefits of tDCS for children with CP. One of the strong points of this study is the use of sham tDCS to effect sham stimulation.

However, this study had some limitations. First, it was a pilot study, and only a small number of children from one center were included. Furthermore, the different number of subjects and different GMFCS levels between the groups may introduce a bias in the comparison. Second, there is no evidence that tDCS actually changed cortical excitability. Studies discussing the effects of tDCS on motor function can measure amplitude changes of motor evoked potentials after tDCS to evaluate excitability changes in the motor cortex (16): however, it is impossible to do so in studies investigating cognition or language. Third, functional imaging studies and EEG were not used in this study to support the results. Fourth, the effect of tDCS only without cognitive training was not investigated in this study. Fifth, the optimal stimulation parameters (e.g., in terms of intensity, duration, polarity, and electrode size) of tDCS cannot be concluded based on this study's finding. Sixth, there was no long-term follow-up assessment to evaluate retention effects. Seventh, some measurements were evaluated by parents of the children, which can be less reliable. Lastly, although the PEDI measures independence in daily living, it is limited in its ability to evaluate daily activities in real life.

## Conclusions

This was the first study investigating the effects of tDCS among children with CP with cognitive impairment. The application of tDCS and cognitive training together was feasible and associated with improvements in cognitive function, ADL, and language among children with CP and cognitive impairment, without severe adverse events. Therefore, tDCS seems to be a promising method for improving cognition in such children. However, the clinical utility of tDCS for children with CP cannot yet be definitively confirmed, and further larger-scale systematic investigation to evaluate the lasting effects and real-world application of tDCS are warranted.

## Data Availability Statement

The raw data supporting the conclusions of this article will be made available by the authors, without undue reservation.

## Ethics Statement

The studies involving human participants were reviewed and approved by Ethical Committee of Asan Medical Center. Written informed consent to participate in this study was provided by the participants' legal guardian/next of kin.

## Author Contributions

MH, EC, and JY were responsible for data collection. EK was responsible for writing the first draft of the article and analyzing the data with MSY. EK and IS revised and finalized the article. All authors contributed to the article and approved the submitted version.

## Conflict of Interest

The authors declare that the research was conducted in the absence of any commercial or financial relationships that could be construed as a potential conflict of interest.

## Publisher's Note

All claims expressed in this article are solely those of the authors and do not necessarily represent those of their affiliated organizations, or those of the publisher, the editors and the reviewers. Any product that may be evaluated in this article, or claim that may be made by its manufacturer, is not guaranteed or endorsed by the publisher.
